# Ribociclib plus endocrine therapy in bone marrow visceral crisis: challenging the chemotherapy paradigm in luminal metastatic breast cancer—a case report

**DOI:** 10.3389/fonc.2025.1669057

**Published:** 2025-12-17

**Authors:** Oscar Ivan Pérez-López, Gonzalo Lendinez-Sanchez, Jesús González-García, Begoña Jiménez-Rodríguez, Myriam Leon-Fradejas, David Fernandez-Garay

**Affiliations:** 1 Department of Medical Oncology, Hospital Regional Universitario de Málaga, Málaga, Spain; 2 Department of Pathology, Hospital Regional Universitario de Málaga, Málaga, Spain

**Keywords:** visceral crisis, metastatic breast cancer, RIGHTCHOICE, ABIGAIL, bone marrow infiltration, luminal breast cancer

## Abstract

**Introduction:**

Visceral crisis in hormone receptor-positive (HR+), HER2-negative metastatic breast cancer poses a therapeutic challenge, traditionally managed with chemotherapy due to the urgency of organ dysfunction. However, recent evidence suggests that cyclin-dependent kinase 4/6 inhibitors (CDK4/6i), in combination with endocrine therapy (ET), may offer rapid and effective disease control even in aggressive presentations.

**Case presentation:**

We report the case of a 52-year-old premenopausal woman diagnosed with *de novo* HR+, HER2-negative metastatic lobular breast cancer presenting with severe anemia and thrombocytopenia secondary to diffuse bone marrow infiltration. The patient was initially managed with letrozole and goserelin, followed by the addition of ribociclib. Clinical and hematologic parameters improved rapidly, and the patient achieved a complete metabolic response after nine treatment cycles without significant toxicity.

**Discussion:**

This case highlights bone marrow infiltration as a form of visceral crisis, reinforcing the heterogeneity of visceral crisis presentations beyond solid organ involvement. The rapid response and sustained disease control observed with CDK4/6i plus ET challenge the traditional paradigm favoring chemotherapy in such scenarios. Supporting evidence from RIGHT Choice, ABIGAIL, and recent case series further validates this therapeutic approach in carefully selected patients.

**Conclusion:**

CDK4/6 inhibitors in combination with endocrine therapy may constitute an effective and well-tolerated alternative to chemotherapy in HR+/HER2− metastatic breast cancer presenting with visceral crisis, including hematologic compromise due to bone marrow involvement. This case underscores the need to reconsider current treatment algorithms to include targeted therapies as a viable and effective option in acute and life-threatening presentations of HR+/HER2− metastatic breast cancer.

## 1 Introduction

Most cases of metastatic breast cancer (MBC) arise from the recurrence of previously treated early breast cancer. However, *de novo* MBC, which presents with metastases at initial diagnosis, accounts for approximately 3% to 6% of new cases in high-income countries (1). In Spain, the 2022 data show similar values, between 5% and 6%. Although the available evidence for premenopausal women is scarce, it is suggested that a significant proportion of young women may present metastatic disease at diagnosis (2).

Cyclin-dependent kinase 4/6 inhibitors (CDK4/6i) such as palbociclib, ribociclib, and abemaciclib have become the standard first-line treatment for hormone receptor-positive (HR+) MBC when used in combination with endocrine therapy (ET). These agents have demonstrated improvements in progression-free survival (PFS) and overall survival (OS) in treatment-naïve patients and are currently recommended as the preferred first-line approach by major clinical guidelines (3, 4).

However, the pivotal trials that led to the approval of these agents excluded patients with symptomatic visceral disease or those at risk of short-term life-threatening complications (5–8). As a result, there is a lack of evidence supporting the use of CDK4/6i in the context of aggressive disease or visceral crisis (VC).

The definition of VC has historically been heterogeneous and inconsistently applied across studies and clinical practice (9–11). Traditionally, chemotherapy has been the preferred approach in such scenarios due to its shorter time to response compared with ET alone (3). However, recent data indicating a significantly reduced time to response with CDK4/6 inhibitors in combination with ET, compared to endocrine monotherapy, have called into question the long-standing paradigm favoring chemotherapy in HR+/HER2− MBC presenting with VC, particularly in patients with imminent organ dysfunction (5–8).

Recent studies have shown that ribociclib, combined with letrozole, provides significant benefits in PFS and OS in patients with visceral metastases (12).

We report the case of a 52-year-old premenopausal woman diagnosed with *de novo* HR+, HER2-negative metastatic lobular breast carcinoma with exclusive bone involvement, who presented acutely severe, life-threatening bone marrow infiltration. The patient was managed successfully with first-line systemic therapy comprising ribociclib in combination with letrozole and goserelin, a gonadotropin-releasing hormone agonist (GnRHa).

## 2 Case presentation

A 52-year-old premenopausal woman, with a family history of breast cancer (paternal uncle at age 70 and maternal aunt at age 50), and no relevant personal medical history, presented in July 2024 with symptoms of fatigue and shortness of breath. A blood test revealed transfusion-range anemia (hemoglobin 4.8 g/dL) and thrombocytopenia (37,000/μL), with a normal white blood cell count. Based on these results, the patient was referred to the emergency department, where blood tests were repeated, confirming severe anemia, thrombocytopenia, and high levels of lactate dehydrogenase (598 U/L) and total bilirubin (1.2 mg/dL). A blood smear was performed, showing 2% schistocytes, dacrocytes, and polychromatophils, confirming the thrombocytopenia.

Clinically, the patient was hemodynamically stable and only experienced shortness of breath while speaking, with no other symptoms.

Given these findings, hematology was consulted due to initial suspicion of autoimmune thrombotic microangiopathy. The patient was admitted to the Intensive care unit (ICU) and started on methylprednisolone 1 mg/kg for 3 days, along with plasmapheresis and transfusion of blood components. She continued daily plasmapheresis, receiving a total of six sessions and transfusions, with a poor response to the treatment.

**Figure 1:**
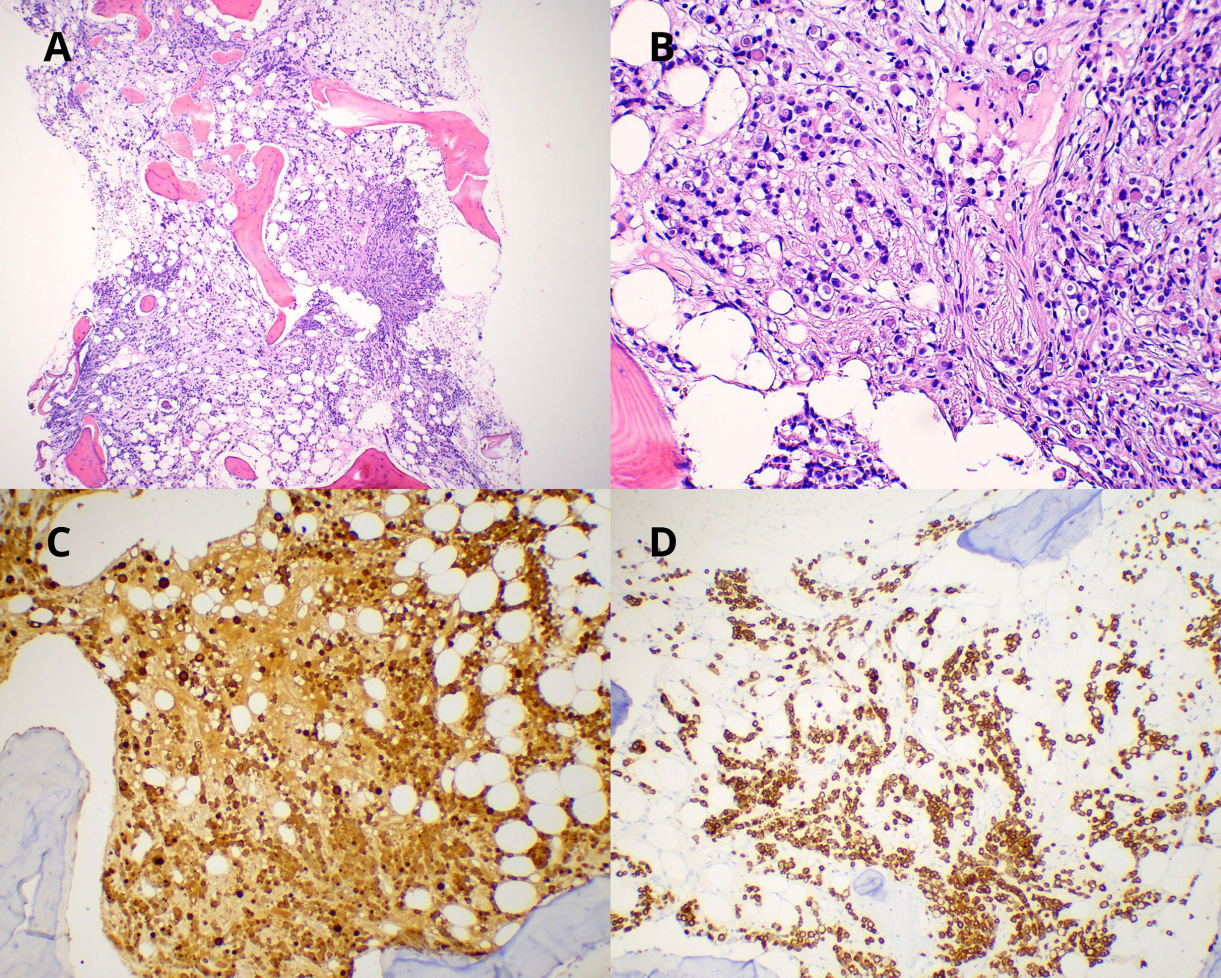
**(A)** Hematoxylin and eosin stain (×5): bone marrow core biopsy showing neoplastic infiltration by lobular breast carcinoma with desmoplastic reaction. **(B)** Hematoxylin and eosin stain (×20): the neoplastic cells are discohesive, small, monomorphic, with mild atypia and signet ring morphology. **(C)** Immunoreactivity for mammaglobin (×20) showing diffuse membranous and cytoplasmic staining in neoplastic cells. **(D)** Immunoreactivity for cytokeratin 7 (×10) showing diffuse cytoplasmic staining in neoplastic cells.

An immunological and autoimmune study was performed, including tests for rheumatoid factor, Antinuclear antibodies (ANAs), anti-native DNA, anticardiolipin antibodies, anti-β2 glycoprotein I antibodies, and lupus anticoagulant, with no relevant findings, and an initial thoraco-abdominopelvic CT scan showed no evidence of distant disease. In the absence of other symptoms or significant imaging findings, a PET–CT scan was requested, showing hypermetabolic lesions in the spine, sternum, clavicles, and bilateral femurs. Concurrent blood work revealed elevated tumor markers [Carcinoembryonic Antigen (CEA), 84 ng/mL; Cancer Antigen 125 (CA 125), 1,873 U/mL; Cancer Antigen 15-3 (CA 15-3), 306 U/mL; Carbohydrate Antigen 19-9 (CA 19.9), 4,071 U/mL], and a bone marrow biopsy showed infiltration by carcinoma of breast origin. Histopathological images of the bone marrow biopsy are shown in **Figure 1**.

Based on these findings, primary occult breast cancer was suspected, and bilateral mammography and breast MRI were performed, identifying a solid lesion with poorly defined margins, likely multifocal, in the lower outer quadrant of the left breast. A core needle biopsy confirmed invasive lobular carcinoma of the breast, with 100% estrogen receptor positivity, 50% progesterone receptor positivity, a 5% Ki67 proliferative index, and HER2 2+ (non-amplified), classifying it as a luminal A subtype.

Following diagnosis, on August 6, 2024, inpatient treatment was initiated with letrozole 2.5 mg daily and goserelin 3.6 mg monthly.

After 1 week of treatment, the patient was discharged due to improvement in hemoglobin (10.6 g/dL) and platelet count (93,000/μL), without the need for further transfusions. On August 22, she attended a medical oncology consultation, where ribociclib 600 mg and denosumab 120 mg monthly were added to the treatment.

**Figure 2:**
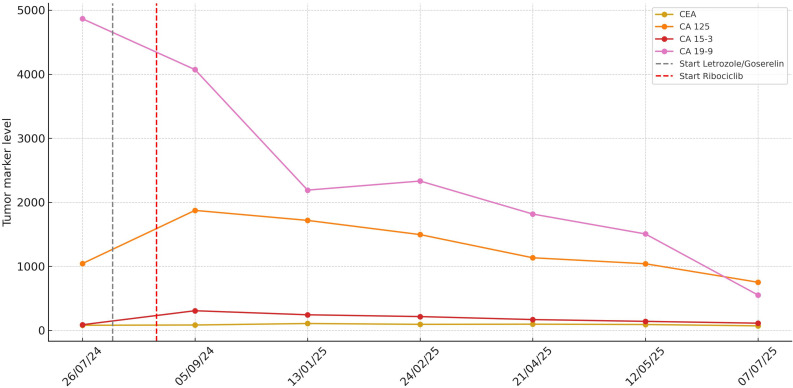
Evolution of tumor markers (CEA, CA 15.3, CA 19.9, and CA 125) since diagnosis and evolution after initiation of systemic treatment.

**Figure 3:**
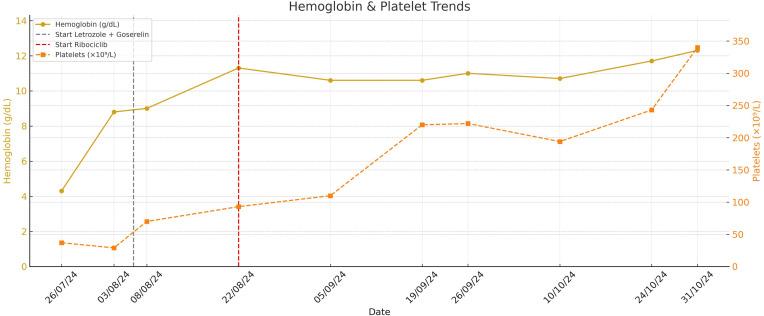
Platelets and hemoglobin levels since diagnosis and evolution after initiation of systemic treatment.

During the first cycle with ribociclib, the patient developed grade 3 neutropenia (920/μL), afebrile, which led to a 1-week delay in treatment and recovery to grade 1, with no further episodes of grade 3 neutropenia. Likewise, after one treatment cycle, platelet count (110,000/μL) and hemoglobin (11.3 g/dL) improved significantly, returning to grade 1 with no associated bleeding events. The timeline of treatment and trends of tumor markers, hemoglobin, and platelets are shown in **Figures 2**, **3**.

**Figure 4:**
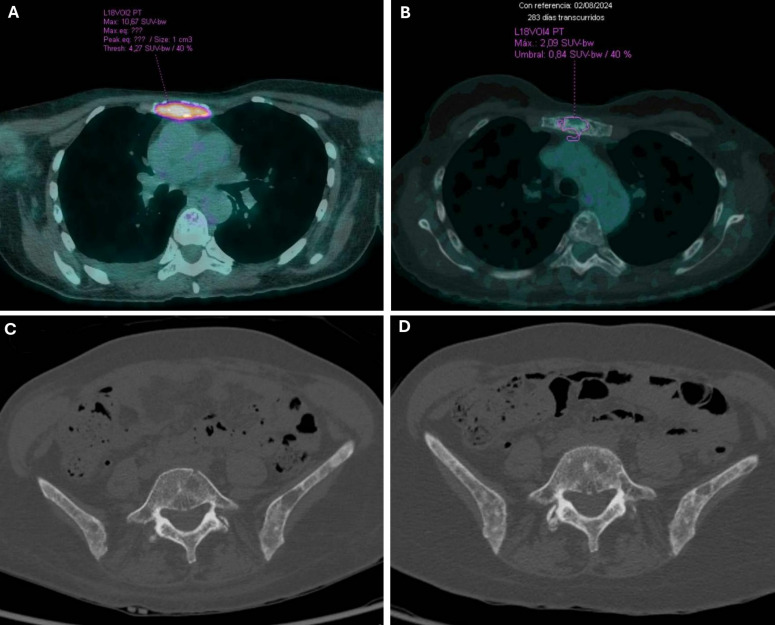
Comparative PET–CT images at diagnosis and after treatment. **(A)** PET–CT 08/02/2024: a lytic lesion was observed in the sternum with high metabolic activity [reaching a Maximum Standardized Uptake Value (SUVmax) of up to 10.67]. **(B)** PET–CT 05/13/2025: changes were observed in the sternum with low metabolic uptake (SUVmax 2.76, previously 20.9). **(C)** PET–CT 08/02/2024: at the pelvic level, there was disruption of the bone architecture with a permeative bone pattern predominantly affecting the sacrum and iliac bones, where hypermetabolic foci associated with sclerotic-lytic lesions were observed (sacrum, SUVmax up to 5.47; iliac wings, SUVmax up to 9.47; ischiopubic rami, SUVmax 5.65). **(D)** PET–CT 05/13/2025: at the bone level, multiple predominantly lytic changes persist in the axial skeleton, without significant metabolic uptake and with no changes compared to previous studies.

In the first reassessment in January 2025, PET–CT showed a significant reduction in metabolic activity of the skeletal involvement, with persistent extensive axial and appendicular lesions of predominantly lytic pattern but with minimal activity. These findings were consistent with a partial morphometabolic response compared to the prior PET scan. Comparative PET–CT images at diagnosis and after treatment are shown in **Figure 4**.

The patient has continued treatment to date, having received a total of nine cycles, with no clinically significant toxicity and maintaining a good quality of life. The most recent reassessment with PET–CT, performed on May 12, 2025, showed a complete metabolic response.

## 3 Discussion

Approximately one-third of newly diagnosed breast cancer cases occur in premenopausal women, in whom the disease often exhibits a more aggressive clinical behavior (3, 12, 13).

The definition of VC remains the same since the 5th International Consensus Conference for Advanced Breast Cancer (ABC 5) (11) with no changes in the 6th or 7th edition (14). VC occurs in approximately 10%–15% of cases of advanced breast cancer (11) and is defined as severe organ dysfunction, as assessed by signs and symptoms, laboratory studies, and rapid progression of disease. It is not the mere presence of visceral metastases but implies important organ compromise leading to a clinical indication for the most rapidly efficacious therapy (11). Some studies have proposed that pancytopenia secondary to bone marrow infiltration, among other causes, may represent a form of VC, particularly when hematopoietic function is so severely impaired that it poses a life-threatening risk to the patient (15). Currently, there are no universally accepted objective clinical criteria to define VC, and different studies have reported a wide range of clinical scenarios encompassed under this term.

In the context of bone marrow infiltration, visceral crisis can be defined by severe anemia [Hemoglobin (Hb) < 7–8 g/dL], significant thrombocytopenia (<50,000/μL), or pancytopenia associated with clinical symptoms (15).

In a review aimed at defining prognosis based on organ dysfunction related to VC, liver involvement was associated with worse OS, whereas bone marrow metastases were associated with better survival rates, reaching 1 year in some cohorts (15).

There is no consensus on the most effective therapy, and new treatments with evidence in this setting are emerging (11, 15–17). Clinical guidelines recommend considering chemotherapy for patients presenting with VC, regardless of hormone receptor status, in order to achieve rapid disease control (15, 16).

We present a clinical case illustrating this concept of VC in a 52-year-old premenopausal woman with HR+, HER2-negative lobular MBC. The patient was treated successfully with a combination of ribociclib, letrozole, and goserelin, instead of the traditionally recommended chemotherapy; notably, hematologic parameters quickly improved in the first cycle of ribociclib.

One aspect to consider in our clinical case is the possible influence on the frequency of VC based on tumor-related factors such as histology (ductal or lobular) and the proliferation index Ki67. Our patient was diagnosed with an invasive lobular carcinoma. There are not enough studies on the incidence of VC according to the histological type of breast cancer, although a retrospective study analyzed histological type and proliferation index in a sample of patients with MBC who presented with VC at diagnosis. This study showed that invasive ductal carcinoma was more frequently associated with VC than invasive lobular carcinoma, likely due to the fact that approximately 92% of the patients in the study had invasive ductal carcinoma, making it an inadequate comparison. A similar situation applies to the proliferation index, where a high Ki67 (>15%) was more strongly associated with VC (16).

Mutational profiling was not performed because the patient had not received prior endocrine therapy, making an ESR1 mutation unlikely. Since PIK3CA status does not influence first-line treatment—CDK4/6 inhibitor plus endocrine therapy—its determination is not proposed until disease progression.

Meta-analyses and randomized trials have demonstrated that the combination of endocrine therapy with CDK4/6 inhibitors provides better outcomes in PFS, OS, and quality of life compared to chemotherapy, including in patients with visceral crisis. The RIGHT Choice and Young-PEARL studies directly compare these strategies in the context of aggressive disease and show that combined endocrine therapy is not only non-inferior but also superior in terms of tumor control and tolerability (25).

To date, there is only one prospective clinical trial published exploring an initial management with CKDi in an aggressive debut. RIGHT Choice was a randomized phase 2 clinical trial of ribociclib plus letrozole plus goserelin that explored the efficacy and safety of this combination versus chemotherapy in pre-/perimenopausal women with clinically aggressive HR+/HER2− advanced breast cancer. In the trial, 47.7% patients had investigator-assessed VC. The median time to response was 4.9 versus 3.2 months with ribociclib plus ET and combination CT, with no significant statistical differences between groups (12). In this study, the data show superior PFS with ribociclib plus ET compared to combination chemotherapy, with similar response rates, a lower incidence of symptomatic adverse events, and fewer treatment discontinuations due to treatment-related toxicity (12).

The ABIGAIL study was a randomized phase II study of abemaciclib plus ET with or without a short course of induction paclitaxel in patients with previously untreated HR-positive/HER2-negative advanced breast cancer with aggressive disease criteria (18). Outcomes were presented as abstracts in the European Society of Medical Oncology (ESMO) 2024 Congress, and they showed a better objective response rate in the first 12 weeks, favoring abemaciclib + ET versus paclitaxel. A better safety and tolerability profile was also demonstrated with abemaciclib and ET compared to chemotherapy (18).

The PADMA trial (palbociclib plus ET versus chemotherapy in high-risk HR+/HER2− patients) demonstrated a clear improvement in progression-free survival (median PFS 18.7 months with palbociclib + ET *vs*. 7.8 months with chemotherapy; HR 0.45) and time to treatment failure, with a numerical trend toward improved overall survival. These larger prospective data reinforce the evidence that CDK4/6 inhibitor-based ET regimens are not only active but also outperform chemotherapy in disease control outcomes among high-risk patients (24).

At a lower level of evidence, retrospective studies, such as the one published by Behrouzi et al., have evaluated the use of CDK4/6 inhibitors (92.6% of patients treated with palbociclib) in combination with ET versus weekly paclitaxel or nab-paclitaxel in patients with VC. The median time to first objective improvement was similar between the groups; however, the disease control rate at 4 months, time to treatment failure, PFS, and OS were significantly better in the CDK4/6i group (19).

In addition, case series and case reports of patients with VC or short-term life-threatening conditions further support the use of CDK4/6i plus ET as initial treatment in this specific clinical context (20–23). Due to similarities with our case, we highlight the case of a 36-year-old premenopausal Javanese woman with liver involvement and disseminated carcinomatosis of the bone marrow (DCBM) secondary to metastatic lobular hormone HR+/HER2-negative breast cancer. The patient presented with severe anemia (hemoglobin 3.6 g/dL), thrombocytopenia (24,000/μL), and markedly elevated lactate dehydrogenase (LDH; 2,616 U/L). She was treated with a combination of ribociclib, leuprorelin, letrozole, and zoledronic acid. Clinical and laboratory improvements were evident within the first two cycles, and she regained normal functional status within 6 months, with no severe toxicities reported (20).

Current data demonstrate that CDK4/6 inhibitors combined with endocrine therapy can induce rapid and clinically meaningful responses, even in situations of visceral crisis and bone marrow involvement (as reviewed in real-world cases and small series). This challenges the classical paradigm and supports considering endocrine therapy as a valid option in visceral crisis, particularly given that our patient’s tumor exhibits a low Ki67 index and a luminal phenotype, indicating high hormonal sensitivity (19, 26).

With CDK4/6 inhibitor-based endocrine therapy, the toxicity profile is much more favorable than that of chemotherapy, with a lower risk of febrile neutropenia, mucositis, alopecia, and other serious complications. This translates into a better quality of life and less impact on our patient’s overall condition. Additionally, it is worth noting that our patient initially presented with bicytopenia, and chemotherapy in this case could have worsened the myelosuppression, whereas hormonal therapy would not, allowing for hematologic recovery and tumor control.

Focusing on our case, after the initiation of endocrine therapy, our patient achieved laboratory stabilization within 7 days and further normalization after the first cycle of ribociclib (approximately 3–4 weeks).

In prospective trials, the median time to objective response with ribociclib was 4.9 months (RIGHT Choice), and the ABIGAIL study showed differences in Objective Response Rate (ORR) at 12 weeks. This suggests that hematologic responses (recovery from bone marrow infiltration) may occur earlier than radiologic responses, and that the combination of ET with CDK4/6 inhibitors can induce rapid functional and hematologic recovery in patients with bone marrow involvement.

Regarding the duration of response or disease control, both the RIGHT Choice and PADMA trials reported prolonged median PFS with CDK4/6 inhibitors compared to chemotherapy (RIGHT Choice, PFS 21.8 *vs*. 12.8 months; PADMA, 18.7 *vs*. 7.8 months). In our case, the patient maintained sustained clinical and radiologic control until the last assessment (complete metabolic response documented between 6 and 7 months, with no clinically significant toxicity after 9 cycles), consistent with the medium-term efficacy observed in these trials.

At this point, this case provides additional clinical evidence that a combination of ET and CDK4/6 inhibitors (ribociclib) can be associated with early and sustained hematologic recovery in patients with bone marrow involvement and hematologic visceral crisis. This finding is relevant because the traditional recommendation for VC typically favors initial chemotherapy for rapid disease control. Our case demonstrates that, at least in some HR+/HER2− patients (luminal A subtype, low Ki67), the ET plus CDK4/6 inhibitor strategy can avoid initial chemotherapy while achieving both urgent clinical stabilization and durable metabolic responses.

Our case, along with the aforementioned reports and the majority of patients included in the RIGHT Choice and ABIGAIL studies, presented with aggressive or visceral crisis-associated HR+/HER2− MBC, and were successfully treated with CDK4/6 inhibitors in combination with ET.

## 4 Conclusions

- The definition of VC remains heterogeneous and should not be limited to visceral organ involvement. Bone marrow infiltration by MBC, leading to severe cytopenia and immediate multiorgan dysfunction, should also be encompassed within this definition.
- We report a case of VC in a premenopausal woman with HR+/HER2-negative lobular MBC successfully treated with a combination of ribociclib, letrozole, and goserelin. Clinical and hematologic improvement was observed within the first 4 weeks of treatment.
- Aggressive or short-term life-threatening HR+/HER2− MBC may be effectively managed with CDK4/6 inhibitors in combination with ET. This approach is generally better tolerated and can achieve rapid clinical responses while providing sustained disease control superior to that of conventional chemotherapy.

## Data Availability

The original contributions presented in the study are included in the article/Supplementary Material. Further inquiries can be directed to the corresponding author.
